# Dementia risk reduction in China: Country‐specific estimates of modifiable risk factors and population attributable fractions (PAFs)

**DOI:** 10.1002/alz.70542

**Published:** 2025-08-07

**Authors:** Yujing Zhou, Jing Liao, Yu‐Tzu Wu

**Affiliations:** ^1^ Department of Medical Statistics School of Public Health Sun Yat‐Sen University Guangzhou PR China; ^2^ Sun Yat‐sen Global Health Institute Institute of State Governance Sun Yat‐sen University Guangzhou PR China; ^3^ Population Health Sciences Institute Faculty of Medical Sciences Newcastle University Newcastle Upon Tyne UK

**Keywords:** China, population attributable fractions, risk factors for dementia

## Abstract

**INTRODUCTION:**

This study updates the population attributable fractions (PAFs) for 12 dementia risk factors in China, categorized as early life (education), midlife (obesity, hypertension, hearing loss, alcohol abuse, and traumatic brain injury), and later life (smoking, depression, social isolation, physical inactivity, air pollution, and diabetes).

**METHOD:**

Relative risks and communality were calculated from literature. The prevalence was estimated using the latest Chinese Health and Retirement Longitudinal Study (CHARLS); other nationwide surveys substitute for lacking CHARLS factors.

**RESULTS:**

The 12 risk factors account for 60.3% of dementia cases in China, including 14.0% in early life, 8.8% in midlife, and 37.5% in later life. Some factors (e.g., alcohol abuse, depression) showed wide confidence intervals indicating lack of evidence.

**DISCUSSION:**

This study highlights the potential for dementia prevention in China, but more evidence is needed to estimate PAFs for specific risk factors (e.g., midlife factors).

**Highlights:**

- We used the most recent and nationally representative data to calculate population attributable fractions (PAFs) for dementia risk factors in China.
- In China, 60.3% of dementia cases were attributed to the 12 modifiable risk factors. Education was estimated to contribute 14.0% of dementia cases, and 37.5% was attributed to later‐life lifestyle and health factors in China.
- It is necessary to consider incorporating China‐specific factors and updating the PAF calculation method.

## 1 INTRODUCTION

Population attributable fraction (PAF)^1^ has been used to assess the public health impact of dementia since the early 2010s. The calculation method is continually improving. The study by Barnes and Yaffe assessed the PAF for Alzheimer's disease (AD) based on seven potentially modifiable risk factors.^2^ The later work by Norton et al. revised the PAF estimates by considering the correlations between the seven risk factors (i.e., communality) and suggested an overall PAF of 30% worldwide, with similar estimates for the United States, Europe, and the UK.^3^ This indicates that three out of ten AD cases could have been attributed to these risk factors.

Built on this approach, the Lancet Commission 2020 report identified 12 modifiable risk factors across the life course, namely, less education in early life; hearing loss, traumatic brain injury (TBI), hypertension, alcohol abuse, and obesity in midlife; as well as smoking, depression, social isolation, physical inactivity, air pollution, and diabetes in later life. This report provided an overall PAF estimate of 40%, indicating that nearly half of dementia cases worldwide can be attributed to these factors.^4^ While this report suggests a positive message for dementia prevention and population health, these global estimates might not inform situations in specific countries. A recent meta‐analysis on the PAFs of risk factors for dementia found that for low‐ and middle‐income countries (LMICs), the overall PAF values range between 44% and 80%.^5^ This finding suggests that there may be more potential for dementia risk reduction in LMICs than their high‐income counterparts. To understand these differences, it is essential to consider the components used to calculate the PAF estimates.

The current PAF calculation is based on relative risk (RR) estimates reported in longitudinal cohort studies, the frequency of a risk factor presenting in a population (i.e., prevalence), and its interrelationships with other risk factors (i.e., communality).^4^ The RR estimate, which indicates the estimated (causal) effect of exposure to a risk factor on dementia risk, needs to be unconfounded when using the Levin approach to estimate unbiased PAF but this is unlikely to be achieved in most studies reporting partially adjusted RR estimates.^6^, ^7^ In addition, the effect of risk factors may be heterogeneous and vary across different countries. For instance, cross‐national studies have documented differences in the protective effects of education on cognitive functioning,^8^ and the incidence of dementia in older age.^9^ These variations may stem from differing mechanisms through which education influences occupational opportunities, lifestyle choices, and health trajectories across the life course in diverse sociocultural contexts, ultimately shaping cognitive health in later life. Similarly, other risk factors, such as social isolation,^10^ air pollution,^11^ and others, may exert different effects on dementia risk depending on the population and contextual environment. Variations may also derive from prevalence and communality of individual risk factors in different populations. An earlier cross‐country comparison study using data from the 10/66 study found the PAF estimates were higher in India, China, and six Latin American countries than the world average and differed from each other.^12^ However, the data for China only came from two districts of Beijing,^13^ which is not nationally representative.

**RESEARCH IN CONTEXT**

1. **Systematic review**: We searched PubMed and Web of Science to identify studies on the population attributable fraction (PAF) of dementia risk factors in China. The PAF values were estimated from 44.7% to 66.8% with 7 to 12 risk factors. There is a need to provide the up‐to‐date PAF estimates using the latest prevalence data and relative risk estimates of dementia from the literature.
2. **Interpretation**: Our findings suggest 60.3% of dementia cases in China could be attributed to 12 risk factors across early life (14%), midlife (9%), and later life (37%). Less education had the highest contribution.
3. **Future directions**: This study affirms the significant potential for dementia prevention and risk reduction in China. Further studies need to consider incorporating China‐specific factors and improving the PAF calculation method.

Studies on the PAF of dementia risk factors in China are accumulating, but they have certain limitations. Earlier survey‐based studies relied solely on cross‐sectional data,^14^, ^15^ and one review study did not weight the PAF in its calculations.^16^ Recently, two studies applied the 12 risk factors from the Lancet Commission report 2020 to the China Health and Retirement Longitudinal Study (CHARLS) to estimate the PAF values for dementia risk factors in China up to 2018.^17^, ^18^ However, CHARLS lacks clinical diagnosis of dementia,^17^ while applying the global RR estimates to China may introduce bias.^18^ Furthermore, the prevalence of dementia risk factors in the post‐COVID era requires further updates.

To inform country‐specific policies and practice for dementia prevention and risk reduction, the aim of this study is to provide the up‐to‐date, nationally representative PAF estimates for older populations in China. We calculated RR estimates based on meta‐analysis of Chinese longitudinal studies with dementia clinical diagnosis, and updated the latest prevalence data of risk factors from nation‐wide cohort and surveys.

## 2 METHODS

We adopted the approaches used in the Lancet Commission Report 2020^4^ and two previous studies^2^, ^3^ to calculate the PAFs of 12 risk factors. We examined the RR or hazard ratio (HR) estimates for the 12 modifiable risk factors listed in the Lancet Commission Report 2020. For each risk factor, a literature search was carried out in PubMed and Web of Science to identify Chinese longitudinal studies which reported RR/HR estimates of the risk factor. The inclusion criteria for our updated literature search were: (1) studies used the definitions of risk factors as outlined in the Lancet Commission 2020 reports, (2) longitudinal studies based on the general population in China, (3) studies using a clinical diagnosis of all‐cause dementia. We excluded studies focused on subgroups (e.g., only men or individuals with mild cognitive impairment), dementia subtypes, or those using self‐reported diagnoses. From the selected Chinese studies, we extracted pooled estimates of RR and HR. For risk factors with two or more Chinese studies, we conducted a random‐effects meta‐analysis to generate pooled estimates for the Chinese population. We searched for the latest meta‐analyses for each factor, if a particular factor lacked Chinese research, we used the meta‐analysis results as a substitute. More detailed information on literature searches and meta‐analyses is provided in the Appendix in supporting information.

We obtained the prevalence of the 12 risk factors using the latest survey of CHARLS (2020). CHARLS is a nationally representative longitudinal survey of individuals aged ≥ 45 in mainland China.^19^ The prevalence of the risk factors was calculated based on the participants in given age groups, namely, 45 to 64 years for midlife risk factors, and ≥ 65 years for later life, and the entire sample for early life as education attainment was unlikely to change over the life course. The sample weights were applied to ensure the national representativeness of the sample. For risk factors not available in the CHARLS 2020 data (hearing loss, alcohol abuse, obesity, air pollution), we used prevalence estimates reported in other nationally representative surveys. Because we were not able to measure the 12 risk factors in the CHARLS 2020, the communality estimates from a previous study^17^ based on the CHARLS 2018 data were used.

Based on the China‐specific RR estimates and the latest prevalence and communality data, we estimated the weighted overall PAF for the Chinese population using the methods established in previous studies. The weighted PAFs for individual risk factors were calculated based on the proportion of the sum of unweighted PAFs.^4^

## 3 RESULTS

Table 1 reports RR estimates in the Lancet Commission Report 2020,^4^ the latest meta‐analysis,^20^, ^21^, ^22^, ^23^, ^24^, ^25^, ^26^, ^27^, ^28^, ^29^, ^30^, ^31^ and our updated review.^32^, ^33^, ^34^, ^35^, ^36^, ^37^, ^38^, ^39^, ^40^, ^41^, ^42^, ^43^, ^44^ RR estimates reported by longitudinal studies in China were identified for eight risk factors but not four midlife factors (i.e., hearing loss, TBI, hypertension, and obesity).^32^, ^33^, ^34^, ^35^, ^36^, ^37^, ^38^, ^39^, ^40^, ^41^, ^42^, ^43^, ^44^ There is only one longitudinal study for alcohol abuse,^34^ depression,^33^ and diabetes.^41^ Details of the included studies are provided in Table S1 in supporting information. The pooled RR estimate for education was 2.3 (95% confidence interval [CI]: 1.7, 3.0), and for later life smoking was 2.6 (95% CI: 1.6, 4.0), social isolation 1.0 (95% CI: 0.9, 1.2), physical inactivity 1.7 (95% CI: 1.4, 2.0), and air pollution 1.5 (95% CI: 0.9, 2.7). Meta‐analysis results are shown in Figure S1 in supporting information.

**TABLE 1:** Relative risk estimates (95% confidence intervals) of 12 dementia risk factors estimated by prior studies.

	Livingston et al. ^4^	The latest meta‐analysis^20^, ^21^, ^22^, ^23^, ^24^, ^25^, ^26^, ^27^, ^28^, ^29^, ^30^, ^31^	Chinese studies^a^,^32^, ^33^, ^34^, ^35^, ^36^, ^37^, ^38^, ^39^, ^40^, ^41^, ^42^, ^43^, ^44^
Early life			
Less education	1.6 (1.3, 2.0)	1.5 (1.3, 1.6)	2.3 (1.7, 3.0)
Midlife			
Hearing loss	1.9 (1.4, 2.7)	1.6 (1.4, 1.9)	
Traumatic brain injury	1.8 (1.5, 2.2)	1.81 (1.5, 2.1)	
Hypertension	1.6 (1.2, 2.2)	1.20 (1.1, 1.4)	
Alcohol abuse	1.2 (1.1, 1.3)	1.0(1.1,1.2)	1.5 (0.4, 4.9)
Obesity	1.6 (1.3, 1.9)	1.2 (0.7, 2.1)	
Later life			
Smoking	1.6 (1.2, 2.2)	1.3 (1.2, 1.5)	2.6 (1.6, 4.0)
Depression	1.9 (1.6, 2.3)	1.9 (1.5, 2.4)	5.4 (1.7, 17.8)
Social isolation	1.6 (1.3, 1.9)	1.2 (1.1, 1.3)	1.0 (0.9, 1.2)
Physical inactivity	1.4 (1.2, 1.7)	1.3 (1.2, 1.3)^b^	1.7 (1.4, 2.0)
Air pollution	1.1 (1.1, 1.1)	1.0 (1.0, 1.1)	1.5 (0.9, 2.7)
Diabetes	1.5 (1.3, 1.8)	1.4 (1.3, 1.5)	1.5 (1.3, 1.8)

*Note*: Null values indicate a lack of research on this factor in China.

^a^ The relative risk value for alcohol abuse, depression, and diabetes were each based on one specific article.

^b^ Calculated by inversing the original estimate.

Table 2 shows the prevalence from the Lancet Commission Report 2020,^4^ CHARLS 2020, and other Chinese nationwide surveys.^45^, ^46^, ^47^, ^48^, ^49^, ^50^, ^51^ Compared to the worldwide estimates, the prevalence estimates from the CHARLS 2020 data were higher in less education, hypertension, obesity, physical inactivity, and diabetes. Lower prevalence was found in hearing loss, TBI, and depression. The communalities based on the CHARLS 2018 data ranged from 1.1% to 99.3%, with particularly high communalities in hearing loss, hypertension, and physical inactivity (i.e., >88% variance shared with other risk factors).

**TABLE 2:** Prevalence (%) and communality (%) of 12 modifiable risk factors of dementia estimated by the Livingston et al. study and CHARLS.

	Prevalence (%)			
	Livingston et al.^4^,^a^	Nationwide survey^45^, ^46^, ^47^, ^48^, ^49^, ^50^, ^51^	CHARLS 2020^b^	Communality^17^ (%)^c^
Early life				
Less education	40.0	50.3	59.9	34.4
Midlife				
Hearing loss	31.7	18.0	18.0	92.8
Traumatic brain injury	12.1	0.6	4.6	1.3
Hypertension	8.9	44.8	33.6	88.2
Alcohol abuse	11.8		11.5	10.4
Obesity	3.4	16.4	16.4	8.2
Later life				
Smoking	27.4	20.9	22.0	72.6
depression	13.2	7.3	6.7	1.1
Social isolation	17.7		6.6	1.3
Physical inactivity	11.0	76.0	66.7	99.3
Air pollution	75.0		69.5	79.9
Diabetes	6.4	25.0	18.8	10.1

*Note*: Null values indicate a lack of research on this factor in China.

Abbreviation: CHARLS, Chinese Health and Retirement Longitudinal Survey.

^a^ CHARLS 2020 lacked information on hearing loss, alcohol abuse, obesity, and air pollution. The prevalence of hearing loss and obesity was substituted with data from other national surveys.^45^, ^47^ The prevalence of alcohol abuse and air pollution was calculated using CHARLS 2018 results.^17^

^b^ Prevalence estimates were based on the 2010 population and the communality estimates were based on the Health Survey for England 2014.^4^

^c^ Calculated from CHARLS 2018.

Table 3 shows that the weighted overall PAFs were estimated to be 60.3% for the 12 risk factors in early life (14.0%), midlife (8.8%), and later life (37.5%). Less education was found to have the largest PAF (14.0%). For midlife risk factors, the PAFs were generally < 4% with the highest in hearing loss (3.1%). For later life risk factors, the range of PAFs varied largely from 0.1% (social isolation) to 9.9% (physical inactivity). Smoking (8.3%), depression (7.5%), and air pollution (8.9%) had relatively large point estimates of PAFs.

**TABLE 3:** Weighted PAF estimates for China (%).

	PAFs from literature			PAFs based on latest Chinese evidence
	Livingston et al.^4^	Mukadam et al.^12^	Chen et al.^17^	
Weighted overall PAF	39.7	39.5	60.1	60.3
Early life				
Less education	7.1	10.8	11.3	14.0
Midlife				
Hearing loss	8.2	3.9	6.4	3.1
Traumatic brain injury	3.4		1.7	1.2
Hypertension	1.9	6.4	7.4	2.0
Alcohol abuse	0.8		0.9	1.6
Obesity	0.7	5.6	3.8	0.9
Later life				
Smoking	5.2	4.2	4.8	8.3
Depression	3.9	0.5	2.5	7.5
Social isolation	3.5	0.7	3.1	0.1
Physical inactivity	1.6	5.8	7.6	9.9
Air pollution	2.3		6.9	8.9
Diabetes	1.1	1.6	3.7	2.8

*Note*: Null values indicate that the study did not report on this risk factor.

Abbreviation: PAF, population attributable fraction.

## 4 DISCUSSION

### 4.1 Summary of findings

In this study, we used the latest information from the literature to calculate PAF estimates of 12 risk factors in China. The weighted overall PAF was estimated to be 60.3%, which suggests that more than half of dementia cases could be attributed to these risk factors. The early‐life risk factor, less education, was found to have the largest PAF (14.0%) among all the risk factors. Compared to the PAFs reported in the literature, our estimates showed a lower contribution of midlife risk factors (8.8%) but a higher contribution of late‐life risk factors (37.5%) in China.

### 4.2 Interpretation of findings

We found a higher overall weighted PAF value compared to the worldwide estimates reported by the Lancet Commission 2020,^4^ in line with recent evidence highlighting higher PAF estimates in LMICs than high‐income countries.^5^ This may suggest that interventions targeting these modifiable risk factors might be more effective in LMICs in which more than two thirds of people with dementia live.^52^

Earlier studies on PAFs in China included nine or fewer risk factors and reported the overall PAF between 47.7% and 66.8%;^14^, ^15^, ^16^ our estimate used 12 risk factors but was within the range. Our results were also similar to research by Chen et al.^17^ using CHARLS 2018, but we found a lower proportion of PAF in midlife. This may be due to inaccurate RR estimates of CHA RLS used by Chen et al.’s study,^17^ or a lack of Chinese longitudinal studies of midlife risk factors for us to synthesize the evidence. Differences also exist between measures implemented in CHARLS 2018 and 2020. Additionally, some factors in our meta‐analysis showed wide confidence interval (e.g., depression and air pollution), indicating more longitudinal studies are needed.

There could be other dementia risk factors, and some may be specific to the Chinese older population. With evidence evolving, new risk factors are continuously being discovered,^4^, ^53^, ^54^ such that the latest Lancet Commission Report 2024 further included low‐density lipoprotein cholesterol and untreated vision loss.^54^ There may also other modifiable risk factors to be found for Chinese older people. For example, a recent study of 17,500 people aged ≥ 65 across multiple regions in China^14^ identified no spouse/unmarried, olfactory decline, and cardiovascular diseases as country‐specific factors in their PAF estimates. Future studies should further explore a China‐specific set of dementia risk factors.

Our study used the PAF calculation method described in the Lancet reports, namely, the Levin formula to estimate PAFs for individual risk factors and generate the overall PAF using the combined method proposed by Barnes and Yaffe^2^ and communality weighting by Norton et al.^3^ While this method has been widely applied to many studies across the world,^5^ its underlying assumptions have seldom been acknowledged and discussed. In Barnes and Yaffe, they specifically indicate that “it (the overall PAF) assumes that risk factors are independent and that an additive relationship exists between them, which is unlikely to be the case with the risk factors under consideration.” While the independence assumption was addressed in Norton et al.^3^ (i.e., communality), the PAF estimate from this method only provides a conservative estimate and the communality estimation approach cannot address the temporality of risk factors and the causal influences of early‐life factors (e.g., less education) on risk factors in later life stages (e.g., smoking). A recent methodology study has developed an alternative approach to investigate the multiplicative and additive effects of different risk factors and suggested that the PAF estimate for the 12 risk factors can be underestimated.^55^

In addition, a causal inference approach is essential in this field as the concept of PAF involves counterfactual scenarios “What if a risk factor can be removed in a population.” The causal roles of any factors in PAF estimation should be carefully justified, clarifying the concepts of predication and explanation (i.e., causal factors) in the risk factors.^56^ The causal inference techniques need to be applied to make clear assumptions (e.g., directed acyclic graphs^57^) and better quantify the causal effects of single exposure^58^ and multiple co‐occurring risk factors across the life course.^59^ In addition to risk factors in early life, future research should also consider intergenerational factors such as wealth, which can affect familial sharing of material resources and individual health in preconception and/or infancy life stages.^60^ These novel approaches will enhance the PAF estimates and inform evidence‐based policies and practices for specific countries.

### 4.3 Strengths and limitations

Using the established methods of estimating PAFs for dementia, we updated evidence on China‐specific RR estimates and incorporated the prevalence and communality data from population‐representative surveys. There were some limitations. Our study only focused on English publications and did not include the Chinese literature. Only a limited number of longitudinal studies had a clinical diagnosis of dementia and could provide evidence on RR estimates. Most of these studies were regional and provided epidemiological evidence on multiple risk factors. These studies tended to report strong effect sizes with wide 95% CIs. Although we would like to estimate the prevalence and communality of risk factors based on the latest wave of CHARLS data, we could not identify some risk factors (e.g., air pollution) and therefore relied on results from a previous study using CHA RLS 2018.^17^ This study used the PAF estimation method based on the Levin formula with adjustment for communality. While this method can account for interrelationships between multiple risk factors and provide the most conservative PAF estimate, it does not address multiplicative and additional effects of co‐existing risk factors.^55^ The assumptions of PAF estimation methods should be examined in future studies. The latest Lancet Commission 2024 report has included two additional risk factors and reclassified several late‐life risk factors (from the 2020 report), such as smoking, depression, physical inactivity, and diabetes, into the midlife period.^54^ Given that most cohort studies in China focused on older age, the existing studies may not be adequate to clarify the roles of risk factors in mid‐ and later life. To align with the updated framework,^54^ future research should enhance measures for midlife risk factors in the Chinese population to provide high‐quality empirical evidence for PAF estimates.

## 5 CONCLUSIONS

Our study suggests 6 in every 10 dementia cases in China may be attributed to the 12 risk factors across the life course. This highlights the great potential for dementia prevention and risk reduction, but more longitudinal studies with a clinical diagnosis of dementia are needed to provide robust evidence for specific risk factors. Future research should further investigate specific risk factors and develop interventions for the older population in China.

## CONFLICT OF INTEREST STATEMENT

The authors declare that they have no conflicts of interest. Author disclosures are available in the supporting information.

## CONSENT STATEMENT

This study used publicly available data, and informed consent was not required.

## Supporting information




**Table S1**. Summary of included studies
**Figure S1**. Results of Meta‐ analysis

Supporting Information
