# Supplementary material for: Dementia risk reduction in China: Country‐specific estimates of modifiable risk factors and population attributable fractions (PAFs)
Source: Alzheimers Dement. 2025 Aug 7;21(8):e70542. doi: 10.1002/alz.70542 (PMC12329568; doi:10.1002/alz.70542)
Supplement: Supplementary file 1 — Table S1. Summary of included studies Figure S1. Results of Meta‐ analysis [file ALZ-21-e70542-s001.docx]

**Dementia risk reduction in China: country-specific estimates of modifiable risk factors and population attribute fractions (PAFs)**

**Appendix**

**Updated literature searches on the 12 risk factors**

**1. Search strategy**

We conducted a search for articles published before February 1, 2024, using the PubMed and Web of Science databases. For each risk factor, we specifically targeted cohort studies from China. For example, for hearing loss, we searched "all fields" using the search terms "hearing" or "hearing loss" or "hearing aids" or "hearing impairment" AND "China" or "Chinese" AND "dementia". Two authors independently screened the articles, and the selection was reviewed by the senior author.

**2. Inclusion/exclusion criteria**

Inclusion criteria were defined to ensure the relevance and comparability of the studies. Specifically, studies needed to: 1) conform to the definitions of risk factors as outlined in Lancet 2020 ^1^, 2) be cohort studies conducted within China, 3) provide a clear and professional diagnosis of all-cause dementia, 4) sample from the general population, and 5) focus on single-factor analyses to ensure clarity in the association between risk factors and dementia. Additionally, included studies were required to report outcomes as Odds Ratios (OR), Relative Risks (RR), or Hazard Ratios (HR). Exclusion criteria were applied to omit studies focusing on specific subgroups such as exclusively men or individuals with mild cognitive impairment. Those employed self-reported diagnoses, concerned specific subtypes of dementia or cognitive impairments were also eliminated.

**3. Screening and full-text review**

Utilizing the above inclusion and exclusion criteria, we initiated the screening with an evaluation of titles and abstracts and reserved articles of ambiguous eligibility for full-text review. The next step involved a detailed assessment of the full texts for these preliminarily included articles, with any uncertainties resolved through collaborative discussion among authors. A final review by a senior author ensured the inclusion of all pertinent studies.

**4. Results**

We carried out literature searches in PubMed and Web of Science, focusing on 12 risk factors for all-cause dementia. We initially identified 1791 records. At the title/abstract screening stage, a large number of studies were excluded: 1368 papers were excluded as they were irrelevant to our research topic; 236 were not cohort studies, 56 did not sample from the general population, 77 lacked a professional diagnosis of all-cause dementia, 11 investigated the mixed effects of multiple factors, 18 did not report outcomes in OR/RR/HR format, 10 were duplicates.

If multiple publications were from the same cohort study, we selected the one specifically focusing on the risk factors. For example, we encountered a study by Ran ^2^ on air pollution, which, despite the existence of newer studies from the same authors and cohort ^3^, was retained due to its unique focus on physical inactivity. If a publication reports multiple risk factors, we obtained all the relative risk estimates for individual factors. For example, the study by Chen ^4^ encompassed three risk factors: less education, social isolation, and smoking.

A subsequent review by a senior author led to the inclusion of an additional study ^5^, bringing the total count of articles incorporated into our review to 13. Supplementary table 1 provides detailed information on the studies included in the analysis. A random effect meta-analyses were performed for each risk factor with two or more studies (less education, smoking, social isolation, physical inactivity, air pollution) to generate the pooled estimate of relative risk. The results are shown in Supplementary figure 1. For obesity, hypertension, hearing loss, and TBI, where no suitable studies were found, we obtained the pooled estimates from the latest meta-analyses.

|  | years of follow-up | Sample size at baseline | Age at baseline | Factors | Criteria of dementia diagnosis* | Relative risk (95% CI) |
| --- | --- | --- | --- | --- | --- | --- |
| Yuan et al. (2016) ^6^ | 1997-2002 | 16921 | ≥ 55 | less education | DSM-Ⅳ | 2.5 (1.2, 5.0) |
| Gu et al. (2021) ^7^ | 2011-2016 | 1511 | ≥ 60 | less education | DSM-Ⅳ | 2.2 (1.2, 4.3) |
| Zhang et al. (1998) ^5^ | 1987-1992 | 3379 | ≥ 65 | less education | DSM-Ⅲ | 2.2 (1.4, 3.5) |
| Chen et al. (2011) ^4^ | 2001-2007 | 3336 | ≥ 60 | less education, smoking, social isolation | AGECAT-GMS | less education: 2.1 (1.0, 4.4); smoking:  2.4 (1.2, 4.7); social isolation: 1.1 (0.5, 2.6) |
| Deng et al. (2006) ^8^ | 2001-2003 | 2632 | ≥ 60 | alcohol abuse | DSM-Ⅲ | 1.5 (0.4, 4.9) |
| Juan et al. (2004) ^9^ | 2001-2003 | 2820 | ≥ 60 | smoking | DSM-Ⅲ | 2.7 (1.6, 5.4) |
| Chen et al. (2008) ^10^ | 2001-2002 | 1736 | ≥ 65 | depression | AGECAT-GMS | 5.4 (1.7, 17.8) |
| Lee et al. (2018) ^11^ | 2005-2012 | 15582 | ≥ 65 | social isolation | ICD-10 | 1.0 (0.9, 1.2) |
| Wu et al. (2020) ^12^ | 2010-2015 | 1648 | ≥ 65 | physical inactivity | DSM-Ⅳ | 1.6 (1.1, 2.3) |
| Ran et al. (2021) ^2^ | 1998-2011 | 57775 | ≥ 65 | physical inactivity | ICD-9 | 1.7 (1.3, 2.1) |
| Tang et al. (2023) ^13^ | 2001-2011 2014-2019 | Anhui cohort: 3336 Zhejiang cohort: 9353 | ≥ 60 | air pollution | AGECAT-GMS | Anhui cohort: 2.1 (1.0, 4.6) Zhejiang cohort: 2.3 (1.1, 4.9) |
| Ran et al. (2021) ^3^ | 1998-2011 | 66820 | ≥ 65 | air pollution | ICD-9 | 1.1 (1.0, 1.1) |
| Wang et al. (2019) ^14^ | 2010-2015 | 3141 | ≥ 60 | diabetes | DSM-Ⅳ | 1.5 (1.3, 1.8) |

**Supplementary table 1.** Summary of included studies

* The basis for physicians diagnosing dementia and the corresponding versions.

DSM: Diagnostic and statistical manual of mental disorders.

AGECAT: Automated Geriatric Examination for Computer Assisted Taxonomy.

GMS: Geriatric Mental State questionnaire

ICD: International Statistical Classification of Diseases

**Supplementary figure 1.** Results of Meta- analysis


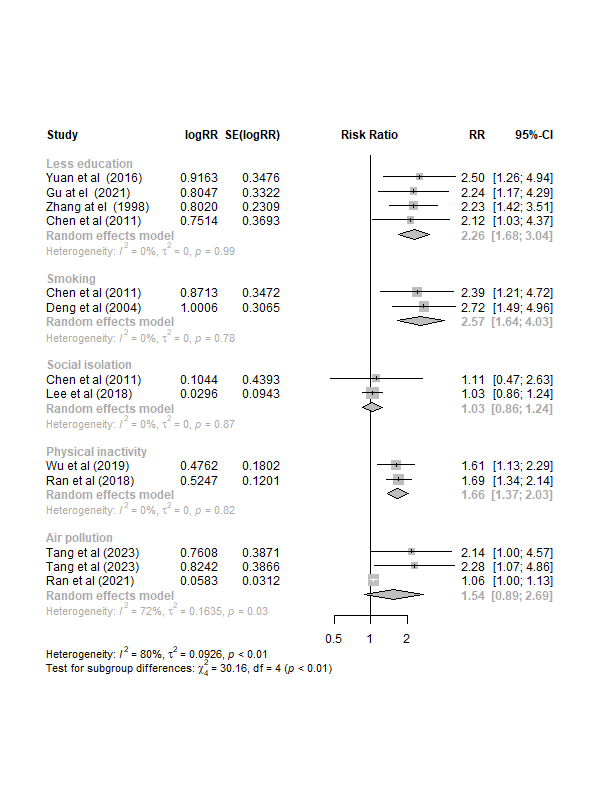


1. Livingston G, Huntley J, Sommerlad A, et al. Dementia prevention, intervention, and care: 2020 report of the Lancet Commission. *Lancet*. Aug 8 2020;396(10248):413-446. doi:10.1016/S0140-6736(20)30367-6

2. Ran JJ, Zhang YM, Han LF, et al. The joint association of physical activity and fine particulate matter exposure with incident dementia in elderly Hong Kong residents. *Environment International*. Nov 2021;156doi:ARTN 106645

10.1016/j.envint.2021.106645

3. Ran JJ, Schooling CM, Han LF, et al. Long-term exposure to fine particulate matter and dementia incidence: A cohort study in Hong Kong. *Environmental Pollution*. Feb 15 2021;271doi:10.1016/j.envpol.2020.116303

4. Chen RL, Hu Z, Wei L, Ma Y, Liu ZM, Copeland JR. Incident Dementia in a Defined Older Chinese Population. *Plos One*. Sep 23 2011;6(9)doi:10.1371/journal.pone.0024817

5. Zhang M, Katzman R, Yu E, Liu W, Xiao SF, Yan H. A preliminary analysis of incidence of dementia in Shanghai, China. Article. *Psychiatry and Clinical Neurosciences*. Dec. 1998;52(SUPPL.):S291-S294. doi:10.1111/j.1440-1819.1998.tb03248.x

6. Yuan J, Zhang ZX, Wen HB, et al. Incidence of dementia and subtypes: A cohort study in four regions in China. *Alzheimers & Dementia*. Mar 2016;12(3):262-271. doi:10.1016/j.jalz.2015.02.011

7. Gu XZ, Shi ZH, Liu S, et al. Incidence and risk factors of dementia and the primary subtypes in northern rural China. *Medicine*. Apr 2 2021;100(13)doi:10.1097/MD.0000000000025343

8. Deng J, Zhou DHD, Li JC, Wang YJ, Gao CY, Chen M. A 2-year follow-up study of alcohol consumption and risk of dementia. *Clinical Neurology and Neurosurgery*. Jun 2006;108(4):378-383. doi:10.1016/j.clineuro.2005.06.005

9. Juan D, Zhou DHD, Li J, Wang JYJ, Gao C, Chen M. A 2-year follow-up study of cigarette smoking and risk of dementia. *European Journal of Neurology*. Apr 2004;11(4):277-282. doi:DOI 10.1046/j.1468-1331.2003.00779.x

10. Chen RL, Hu Z, Wei L, Qin X, McCracken C, Copeland JR. Severity of depression and risk for subsequent dementia: cohort studies in China and the UK. *British Journal of Psychiatry*. Nov 2008;193(5):373-377. doi:10.1192/bjp.bp.107.044974

11. Lee ATC, Richards M, Chan WC, Chiu HFK, Lee RSY, Lam LCW. Association of Daily Intellectual Activities With Lower Risk of Incident Dementia Among Older Chinese Adults. *Jama Psychiatry*. Jul 2018;75(7):697-703. doi:10.1001/jamapsychiatry.2018.0657

12. Wu WQ, Ding D, Zhao QH, et al. Medium-to-High Late-Life Physical Activity Is Associated with Lower Risk of Incident Dementia: The Shanghai Aging Study. *Journal of Alzheimers Disease*. 2020;73(2):751-758. doi:10.3233/Jad-190937

13. Tang J, Chen ATY, He F, et al. Association of air pollution with dementia: a systematic review with meta-analysis including new cohort data from China. *Environmental Research*. Apr 15 2023;223doi:10.1016/j.envres.2022.115048

14. Wang F, Luo JF, Ding D, et al. Elevated Fasting Blood Glucose Level Increases the Risk of Cognitive Decline Among Older Adults with Diabetes Mellitus: The Shanghai Aging Study. *Journal of Alzheimers Disease*. 2019;67(4):1255-1265. doi:10.3233/Jad-180662

**Reference**
